# Time-updated Prediction of Discharge Functional Independence in Patients with Stroke and Low Functional Independence Measure in a Convalescent Rehabilitation Ward: Comparison between Admission and 3-week Models

**DOI:** 10.1298/ptr.25-E10391

**Published:** 2026-06-04

**Authors:** Koichi IKEUE, Keisuke FUNABIKI, Yuya NAKATANI

**Affiliations:** Department of Rehabilitation, Tsukazaki Hospital, Social Medical Corporation Saneikai, Japan

**Keywords:** Patients with stroke and low Functional Independence Measure at admission, Convalescent rehabilitation ward, Time-updated prediction, Prediction model

## Abstract

**Objectives:**

This study aimed to develop 2 multiple linear regression models to predict the Functional Independence Measure (FIM) motor score at discharge in patients with stroke and low FIM at admission (total FIM score ≤55) admitted to a convalescent rehabilitation ward, and to compare their predictive accuracy and internal validity. Model 1 used admission assessments only, whereas Model 2 combined admission assessments with reassessment information at 3 weeks after admission.

**Methods:**

Participants were 104 patients with stroke admitted with a total FIM score of ≤55. The outcome was the discharge FIM motor score (continuous variable). Candidate predictors included basic information and measures of physical and cognitive function. Models were developed using multiple linear regression, and internal validity was evaluated through bootstrap resampling. In Model 1, predictors were age, days since onset, the Berg Balance Scale, and admission FIM motor and cognitive scores. In Model 2, predictors were age, days since onset, admission FIM cognitive score, and the FIM motor score at 3 weeks after admission.

**Results:**

Model 2 showed a higher adjusted R^2^ (0.71) and smaller prediction errors than Model 1 across the evaluated error metrics, indicating superior predictive accuracy. The calibration of Model 2 was also improved compared with Model 1 based on internal validation.

**Conclusions:**

Incorporating reassessment information at 3 weeks improved time-updated prediction of discharge activities of daily living outcomes in this single-center cohort. However, given limited generalizability, external validation using multicenter data (and, if necessary, model updating) is required before broader clinical application.

## Introduction

Stroke remains one of the leading causes of death globally and is a representative disease that leads to the need for long-term care and decline in activities of daily living (ADL)^1)^. Among patients admitted to a post-acute rehabilitation ward, those with substantial ADL disability at admission frequently have difficulty establishing a clear outlook regarding the amount of assistance required and reconstruction of daily life after discharge, making it challenging to plan early interventions and coordinate discharge support. The Functional Independence Measure (FIM) is widely used as a comprehensive index to assess ADL independence. Particularly, the FIM motor score is an important indicator associated with the amount of assistance required at discharge and the likelihood of home discharge^2)^.

In this study, to clarify the characteristics of the target population, patient background was not categorized solely by neurological severity (e.g., National Institutes of Health Stroke Scale: NIHSS) but was instead defined in terms of “functional severity” at admission. In clinical practice in post-acute rehabilitation wards, the daily life function assessment or total FIM score is sometimes used to stratify patients with substantial ADL disability at admission, and previous studies have adopted criteria, including a daily life function assessment score of ≥10 or a total FIM score of ≤55^3–6)^. In this study, we adopted a total FIM score of ≤55 as an operational definition to identify a population with low ADL independence at admission (the “low-FIM-at-admission group”), rather than to define neurological severity.

Furthermore, large-scale overseas data have shown that a low admission FIM score (<60) markedly increases the risk of discharge to destinations other than home^7)^, suggesting that low FIM at admission can be positioned as an indicator reflecting the severity of functional impairment directly linked to discharge outcomes.

Recent studies on FIM prediction in post-acute rehabilitation wards have reported multiple regression models using age, days since onset, physical function assessments, and FIM (motor and cognitive), demonstrating some utility for early support planning^8–10)^. However, in models developed using populations with varying severity, prediction errors may be relatively larger in patients with low admission FIM because of score distribution bias (floor effects) and heterogeneity in recovery trajectories, which can make clinical estimation difficult in discharge planning. Therefore, rather than developing a “general-purpose model,” this study focuses on the low-FIM-at-admission group—whose post-discharge assistance needs and daily life reconstruction are difficult to predict—aiming to improve the practical utility of predictions to support discharge-related decision-making. Conversely, it has been noted that a single assessment at admission may not sufficiently capture individual differences in recovery processes, and dynamic prediction incorporating longitudinal changes (time-updated prediction) has attracted attention^11–16)^. Particularly, 6–10 weeks after stroke onset is considered a period of active spontaneous recovery and neuroplastic changes^17)^, and incorporating reassessment information obtained several weeks after admission into the model may improve the predictive accuracy of ADL outcomes at discharge.

Additionally, for the clinical implementation of prediction models, internal validation to reduce overfitting and ensure reproducibility is important^18)^. The bootstrap method is recommended as an internal validation approach that can be applied even with a limited sample size; however, it should be noted that estimates may become unstable when samples are biased or the sample size is small, and that bootstrap validation does not guarantee external validity^19,20)^. Reports in the field of post-acute rehabilitation remain limited.

Based on these considerations, this study aimed to develop an admission model (Model 1) and a model incorporating reassessment at 3 weeks after admission (Model 2) to predict the FIM motor score at discharge in patients with stroke and low FIM at admission (total FIM score ≤55) admitted to a post-acute rehabilitation ward, and to compare predictive accuracy and internal validity using the bootstrap method.

## Methods

### Participants

The participants were 115 patients with first-ever stroke and hemiparesis who were admitted to the convalescent rehabilitation ward of our hospital between November 2019 and October 2024 and had a total FIM score of ≤55 at admission. Of these, 3 patients who were transferred to an acute-care hospital because of comorbid conditions or recurrent stroke, 4 who were transferred to another convalescent rehabilitation ward, 2 who were discharged within 3 weeks after admission, and 2 with missing data were excluded, leaving 104 patients for analysis.

### Study design and variables

This was a retrospective longitudinal cohort study that followed patients from admission to discharge. Basic information and rehabilitation assessments were retrospectively collected from the electronic medical records (Table 1).

**Table 1.** Baseline characteristics and assessment results of the participants

Variable	Total (n = 104)
Age (years)	76.2 ± 12.2
Sex (n)	Male 56/Female 48
Stroke type (n)	Cerebral infarction 65/Cerebral hemorrhage 39
Pre-admission mRS (score)	0.6 ± 1.1
Days from onset to admission (days)	21.3 ± 15.5
Length of stay (days)	78.9 ± 32.0
Sit-up ability at admission (n)	Independent 19/Assisted 85
FMA upper extremity (score)	20.5 ± 22.0
FMA lower extremity (score)	14.6 ± 10.3
BBS (score)	6.7 ± 11.5; median [IQR] 3 [0–7]; BBS 0–5: 71 (68.3%)
FIM motor score at admission (score)	21.6 ± 7.3; median [IQR] 21 [15–26]; range 13–41
FIM cognitive score at admission (score)	14.3 ± 5.9
FIM total score at admission (score)	35.9 ± 11.0
FIM motor score at 3 weeks (score)	28.7 ± 14.0; median [IQR] 25 [17–37]; range 13–74
FIM cognitive score at 3 weeks (score)	16.5 ± 6.2
FIM total score at 3 weeks (score)	45.2 ± 17.8
FIM motor score at discharge (score)	42.4 ± 21.0; median [IQR] 41.5 [23–58.5]; range 13–84
FIM cognitive score at discharge (score)	20.5 ± 8.3
FIM total score at discharge (score)	62.9 ± 26.9

Values are expressed as mean ± standard deviation or number of cases. For BBS, additional distribution indices are provided to indicate a potential floor effect. For key FIM motor scores, median [IQR] and range are additionally provided to describe the distribution.

mRS, modified Rankin Scale; FMA, Fugl–Meyer assessment; BBS, Berg Balance Scale; FIM, Functional Independence Measure; IQR, interquartile range

The dependent variable was the FIM motor score at discharge, which was treated as a continuous variable.

Ten candidate variables were prespecified for each model. Basic information included age, stroke type (ischemic or hemorrhagic), days since onset, and the premorbid modified Rankin Scale (mRS). Premorbid function was adjusted using the premorbid mRS, which was available for all patients and is widely used internationally; long-term care certification and related indices were not included as major covariates because of concerns regarding the uniformity of records and international comparability. Admission assessments included bed mobility (sit-up ability: independent/dependent), the Fugl–Meyer Assessment (FMA) upper extremity motor score, the FMA lower extremity motor score, the Berg Balance Scale (BBS), the FIM cognitive score, and the FIM motor score. Sit-up ability, which reflects trunk function, was scored with reference to the sit-up item in the “Daily Life Function Assessment” defined by the Ministry of Health, Labour and Welfare in Japan^21)^. A series of movements from the supine position to sitting up on the bed was defined as “sit-up.” Regardless of the use of bed rails or bars, patients who could perform the task independently were classified as “independent” (dummy variable: 1), whereas those who required assistance were classified as “dependent” (dummy variable: 0).

For Model 1, the FIM motor score at admission was entered, whereas for Model 2, the FIM motor score at 3 weeks after admission was entered. The FIM motor score is structurally close to the outcome (FIM motor score at discharge), and including it as an explanatory variable may increase the risk of overfitting. However, because this study aimed to develop a model that emphasizes predictive accuracy and practicality for immediate use in clinical settings, we included it as an explanatory variable (this point is described as a limitation below).

### Statistical analysis

For statistical analysis, we first confirmed the ratio of the number of observations to the number of candidate variables (observations per variable [OPV] = N/k). The sample size in this study was 104, and the number of candidate variables was 10; therefore, OPV was 10.4. This satisfied the commonly recommended criterion (OPV ≥10)^22,23)^, indicating that the number of variables was not excessive relative to the sample size. Additionally, we calculated OPV based on the number of variables selected in the final models (Model 1: k = 5, OPV = 20.8; Model 2: k = 4, OPV = 26.0) to confirm that model complexity was not excessive.

To develop the prediction models, we used multiple linear regression, considering the potential influence of multicollinearity and aiming to clearly present the contribution of each explanatory variable and ensure clinical interpretability^24)^. The 10 candidate variables were prespecified based on previous studies and clinical plausibility as factors reported to be associated with the prediction of ADL at discharge (FIM) in patients with stroke in a convalescent rehabilitation ward and as measures routinely assessed and used for discharge support in clinical practice (Table 1). Variable selection was performed using the stepwise method to obtain a parsimonious model within the prespecified candidate variables, with criteria for entry and removal set at P <0.05. However, because the stepwise method is highly data-dependent and may yield unstable selected variables and estimated coefficients, and has been reported to introduce coefficient bias and overfitting, we additionally performed penalized regression (least absolute shrinkage and selection operator [LASSO]^25)^ and ridge^26)^) as sensitivity analyses to examine the robustness of the estimates.

In the Model 1, the FIM motor score at admission was included as a key explanatory variable, whereas in the Model 2, the FIM motor score at 3 weeks after admission was included as a key explanatory variable; the other candidate variables were common to both models. We constructed the multiple regression equations for each model (Tables 2 and 3) and calculated the coefficient of determination (R^2^) and adjusted R^2^ as performance metrics. Generally, an R^2^ of ≥0.5 (ideally ≥0.7) is considered to indicate a level of explanatory power sufficient for clinical application^12,24)^. Additionally, to assess multicollinearity, we calculated the variance inflation factor (VIF) and confirmed that all variables had VIF values <10.

**Table 2.** Results of multiple regression analysis for the admission model

Variable	Unstandardized coefficient (B)	Standardized coefficient (β)	t Value	P value	95% Confidence interval	VIF
Constant	64.36	—	6.59	<0.001	44.97 to 83.75	—
Age	−0.68	−0.40	−6.07	<0.001	−0.90 to −0.46	1.04
Days from onset to admission	−0.28	−0.21	−3.17	0.002	−0.46 to −0.11	1.06
BBS	0.36	0.20	2.56	0.012	0.08 to 0.64	1.45
FIM motor score at admission	0.82	0.28	3.40	<0.001	0.34 to 1.29	1.67
FIM cognitive score at admission	1.12	0.31	4.44	<0.001	0.62 to 1.62	1.20

Coefficient of determination (R^2^): 0.60; adjusted R^2^: 0.58; p <0.001.

VIF, variance inflation factor; BBS, Berg Balance Scale; FIM, Functional Independence Measure

**Table 3.** Results of multiple regression analysis for the 3-week assessment model

Variable	Unstandardized coefficient (B)	Standardized coefficient (β)	t Value	P value	95% Confidence interval	VIF
Constant	52.56	—	6.31	<0.001	36.02 to 69.10	—
Age	−0.53	−0.31	−5.68	<0.001	−0.71 to −0.34	1.02
Days from onset to admission	−0.22	−0.16	−2.92	0.004	−0.37 to −0.07	1.07
FIM cognitive score at admission	0.57	0.16	2.62	0.010	0.14 to 1.01	1.31
FIM motor score at 3 weeks	0.92	0.61	9.68	<0.001	0.73 to 1.11	1.41

Coefficient of determination (R^2^): 0.72; adjusted R^2^: 0.71; P <0.001.

VIF, variance inflation factor; FIM, Functional Independence Measure

As assumptions of multiple linear regression, we examined the distribution of residuals (Q–Q plot), homoscedasticity (residuals vs. fitted plot), and influential points. The dependent variable (FIM motor score at discharge) had a median (interquartile range [IQR]) of 41.5 [23–58.5] (range, 13–84), and we confirmed that the distribution was not markedly skewed. The bootstrap method is an internal validation approach based on nonparametric resampling and does not require the dependent variable to follow a normal distribution. The distribution of the dependent variable (FIM motor score at discharge) was also examined using a histogram, and considering that distributional bias, including floor effects, could influence estimation, bootstrap resampling was used to evaluate uncertainty in model performance metrics.

To evaluate internal validity for each model, we performed bootstrap validation with 1000 resamples. By repeatedly developing the models in bootstrap samples drawn from the original dataset, we calculated the apparent line and bias-corrected line and constructed calibration plots (Figs. 1 and 2). The agreement with the ideal line (y = x) was visually assessed based on the relationship between predicted (x-axis) and observed (y-axis) values. While the bootstrap method is useful for estimating optimism within the same dataset, we noted that estimates may become unstable when the sample is biased or the sample size is limited, and that bootstrap validation does not guarantee external validity (generalizability to other institutions or periods).

**Fig. 1.**
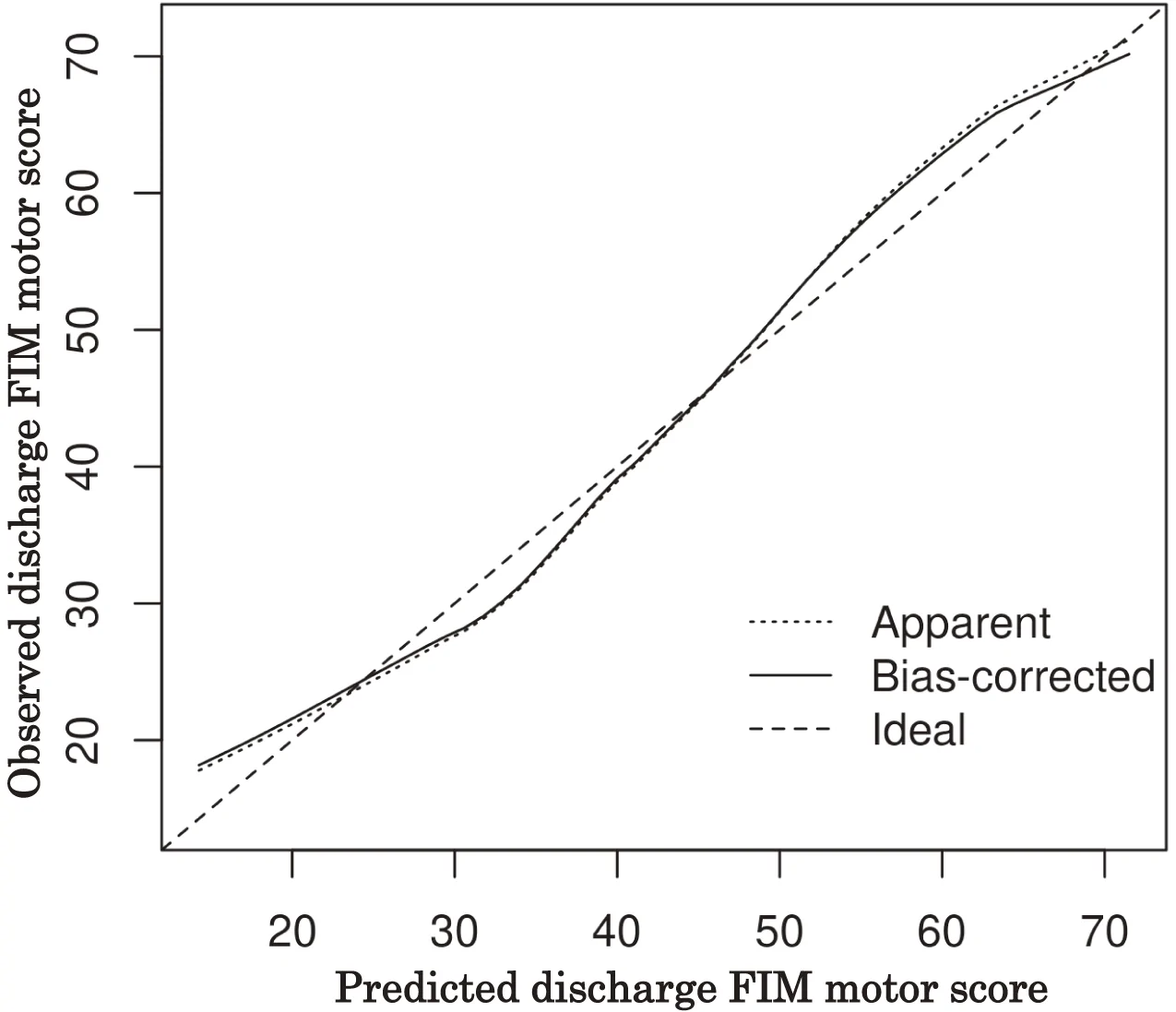
Calibration plot of the admission model (Model 1). The x-axis represents the predicted discharge FIM motor score, and the y-axis represents the observed score. The solid line indicates the apparent fit, the dashed line indicates the bias-corrected fit derived from 1000 bootstrap resamples, and the 45° line represents the ideal reference. FIM, Functional Independence Measure

**Fig. 2.**
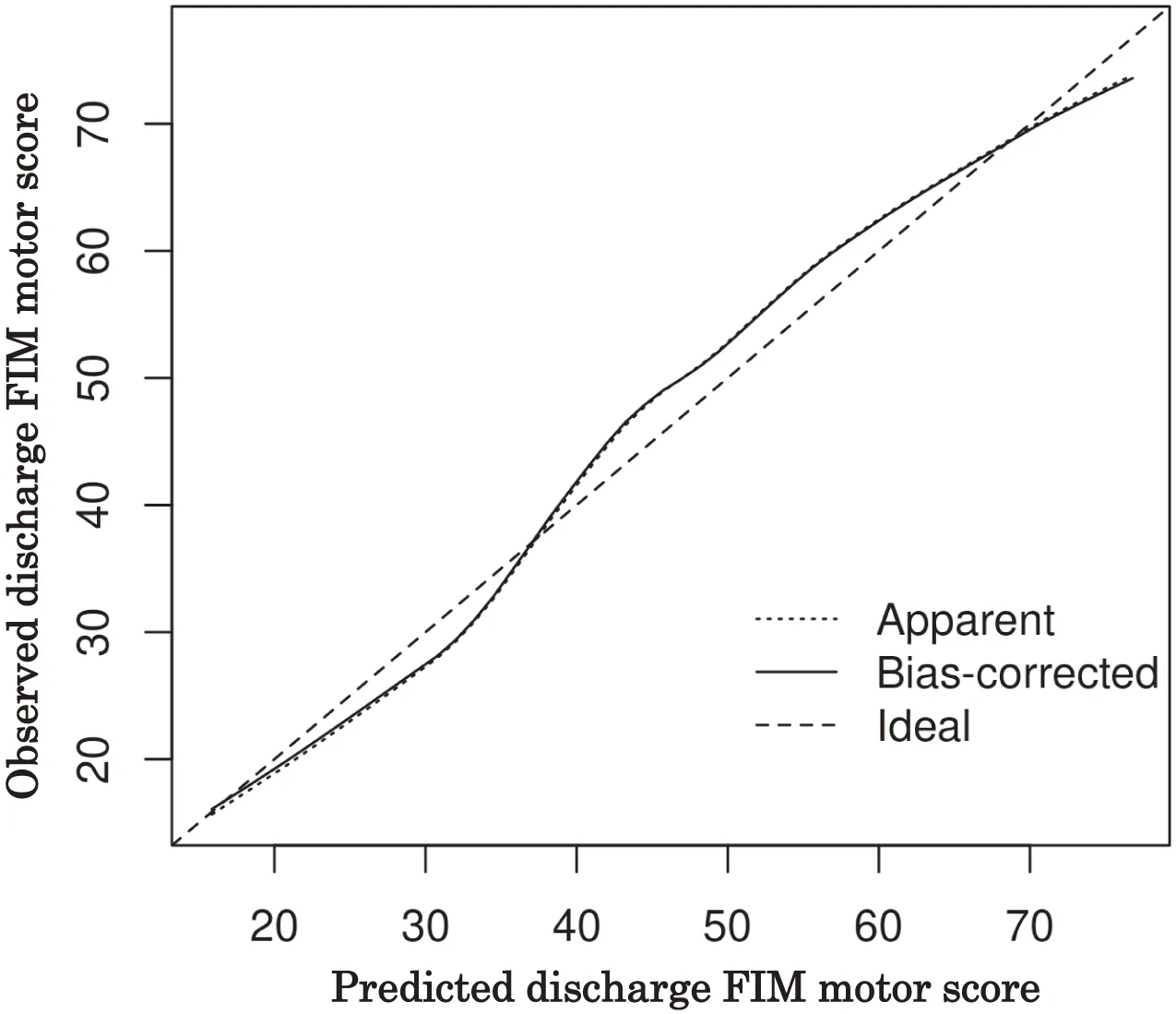
Calibration plot of the 3-week model (Model 2). The x-axis represents the predicted discharge FIM motor score, and the y-axis represents the observed score. Both the apparent and bias-corrected lines show good agreement with the ideal reference line, indicating favorable calibration accuracy for Model 2. FIM, Functional Independence Measure

Furthermore, to comprehensively compare model performance, we calculated the mean absolute error (MAE), root mean square error (RMSE), Akaike information criterion (AIC), Bayesian information criterion (BIC), calibration slope, calibration intercept, and the observed/expected ratio (O/E ratio) (Table 4). Confidence intervals (CIs) were estimated using bootstrap resampling to assess model stability.

**Table 4.** Comparison of internal validity between the 2 models (based on 1000 bootstrap samples)

Index	Admission model (95% CI)	3-Week assessment model (95% CI)
MAE	10.67 (9.13 to 12.41)	8.67 (7.32 to 10.19)
RMSE	12.94 (11.34 to 14.50)	10.85 (9.42 to 12.34)
AIC	841.22 (814.16 to 865.40)	802.55 (773.74 to 829.79)
BIC	859.73 (832.68 to 883.91)	818.42 (789.60 to 845.66)
Calibration intercept	0.00 (−3.90e−14 to 1.67e−14)	0.00 (−3.34e−14 to 1.11e−14)
Calibration slope	1.00 (NA)	1.00 (NA)
O/E ratio	1.00 (NA)	1.00 (NA)
Coefficient of determination (R^2^)	0.61 (0.50 to 0.71)	0.73 (0.64 to 0.80)
Adjusted R^2^	0.59 (0.48 to 0.69)	0.71 (0.62 to 0.79)

Because the calibration slope and O/E ratio were constant at 1.00 across 1000 bootstrap resamples (i.e., zero variance), the 95% confidence intervals could not be estimated (NA).

CI, confidence interval; MAE, mean absolute error; RMSE, root mean square error; AIC, Akaike information criterion; BIC, Bayesian information criterion; O/E ratio, observed/expected ratio; NA, not available

Additionally, as sensitivity analyses, we applied LASSO (α = 1) and ridge (α = 0) regression. After standardizing the explanatory variables, the regularization parameter λ was determined through 10-fold cross-validation (value of λ that minimizes the cross-validated error [λmin] and largest value of λ within 1 standard error of the minimum [λ1SE]). Cross-validated MAE, RMSE, and R^2^ were calculated, and we examined whether the comparison results between Models 1 and 2 were reproduced after changing the analytical approach (Table 5 and Supplementary Table S1).

**Table 5.** Regularized regression (10-fold cross-validation)

Method	λ	Model 1 MAE	Model 1 RMSE	Model 1 R^2^	Model 2 MAE	Model 2 RMSE	Model 2 R^2^
LASSO	λ1SE	12.4	14.7	0.510	10.5	12.5	0.646
LASSO	λmin	11.7	14.0	0.554	9.4	11.7	0.688
Ridge	λ1SE	12.3	14.7	0.510	10.4	12.6	0.635
Ridge	λmin	11.7	14.0	0.552	9.9	12.0	0.672

λmin is the value of λ that minimizes the cross-validated error, and λ1SE is the largest value of λ within 1 standard error of the minimum (a more parsimonious model).

Predictors were standardized, and performance metrics were computed from out-of-fold predictions using the same 10-fold split for all models and methods.

MAE, mean absolute error; RMSE, root mean square error; LASSO, least absolute shrinkage and selection operator; SE, standard error

All statistical analyses were performed using R version 4.4.0 (R Foundation for Statistical Computing, Vienna, Austria), and the significance level was set at 5%.

### Ethical considerations

This was a retrospective observational study that utilized anonymized medical record information of patients admitted to our convalescent rehabilitation ward. At admission, patients were informed that their anonymized clinical data might be used for research purposes, and comprehensive written informed consent for research use was obtained in advance. Therefore, an opt-out consent procedure was not implemented. The study was conducted in accordance with the principles of the Declaration of Helsinki and was approved by the Tsukazaki Hospital Ethics Committee (approval number: 2507003).

## Results

The participants’ baseline characteristics and assessment results are presented in Table 1. The mean age was 76.2 ± 12.2 years, and the number of days since onset was 21.3 ± 15.5 days. The stroke types were ischemic and hemorrhagic stroke in 65 and 39 patients, respectively.

Two multiple linear regression models were developed with the FIM motor score at discharge as the dependent variable (Tables 2 and 3). In the Model 1, the regression equation was as follows: FIM motor score at discharge = 64.36 + age × (−0.68) + days since onset × (−0.28) + BBS × (0.36) + FIM motor score at admission × (0.82) + FIM cognitive score at admission × (1.12) (P <0.01). The model had an R^2^ of 0.60 and an adjusted R^2^ of 0.58. In the Model 2, the regression equation was as follows: FIM motor score at discharge = 52.56 + age × (−0.53) + days since onset × (−0.22) + FIM cognitive score at admission × (0.57) + FIM motor score at 3 weeks after admission × (0.92) (P <0.01). The R^2^ was 0.72, and the adjusted R^2^ was 0.71. The 95% CI for each performance metric was estimated using bootstrap resampling (1000 resamples). Consequently, the adjusted R^2^ was 0.59 (95% CI, 0.48–0.69) and 0.71 (95% CI, 0.62–0.79) for Models 1 and 2, respectively, indicating greater stability and explanatory power for Model 2. The OPV values were 20.8 and 26.0 for Models 1 and 2, respectively, meeting Harrell’s criterion (OPV ≥approximately 20 is desirable)^22)^.

Table 4 presents the bootstrap validation results for each model. The 3-week model had an MAE and RMSE of 8.67 and 10.85, respectively, which were smaller than those of the admission model (10.67 and 12.94, respectively). The AIC and BIC were also lower (802.55 and 818.42, respectively), indicating a better fit. The calibration intercept was close to 0, and because the calibration slope and O/E ratio were consistently 1.00 across all bootstrap resamples, the 95% CIs could not be estimated (NA). Overall, both models showed good calibration accuracy. These results suggest that the Model 2 has smaller errors and superior stability and reproducibility.

As sensitivity analyses, penalized regression (LASSO and ridge) was performed, and predictive performance was compared using 10-fold cross-validation (Table 5). With LASSO regression (λ1SE), MAE, RMSE, and R^2^ were 12.43, 14.65, and 0.510 for Model 1, respectively, whereas they were 10.47, 12.46, and 0.646 for Model 2, respectively. Model 2 also showed better performance at λmin (MAE 9.40, RMSE 11.70, and R^2^ 0.688), and a similar trend was observed for ridge regression (λ1SE: MAE 12.33/RMSE 14.65/R^2^ 0.510 for Model 1 and MAE 10.38/RMSE 12.64/R^2^ 0.635 for Model 2).

The predictors selected via LASSO regression (λ1SE) were premorbid mRS, FIM cognitive score at admission, FIM motor score at admission, age, BBS, days from onset to admission, and FMA lower extremity for Model 1, and FIM motor score at 3 weeks after admission, premorbid mRS, age, FIM cognitive score at admission, and days from onset to admission for Model 2 (Supplementary Table S1).

Calibration plots are shown in Figures 1 and 2. In each figure, the x- and y-axis indicate the predicted FIM motor score at discharge and the observed value, respectively, and agreement with the 45° line (ideal line) was visually compared. Both models showed small discrepancies between observed and predicted values and demonstrated good fit; however, the admission model tended to overestimate values in the 30–40 range and underestimate values above 50. The 3-week model showed overall good agreement, with a tendency toward overestimation below 35 and underestimation above 40.

## Discussion

### Characteristics and significance of the predictive models

A notable feature of this study is that we developed 2 prediction models for ADL at discharge in patients with stroke and low FIM at admission (total FIM score ≤55) who were admitted to a post-acute rehabilitation ward, using assessment information at admission and at 3 weeks after admission, and comprehensively compared their predictive accuracy and internal validity. Both models demonstrated a certain level of explanatory power and accuracy in predicting the FIM motor score, and in particular, Model 2 showed better performance regarding R^2^, MAE, RMSE, AIC, and BIC, highlighting the value of reassessment.

The adjusted R^2^ of 0.71 in this study was comparable to that reported by Tokunaga and Sannomiya^10)^ (adjusted R^2^ = 0.694), indicating that the model has sufficient explanatory power for predicting outcomes in post-acute rehabilitation. In bootstrap validation, the calibration intercept was close to 0, and the calibration slope and O/E ratio were consistently 1.00 across all resamples; consequently, the 95% CIs were not applicable because of zero variance, indicating stable calibration. These findings suggest that adding reassessment at 3 weeks after admission may improve the predictive accuracy for ADL at discharge.

However, the clinical significance of the difference in R^2^ (approximately 0.12) should be evaluated not only by the increase in explained variance but also by the extent to which prediction error is reduced. In this study, compared with Model 1, Model 2 showed a reduction of approximately 2 (10.67–8.67) and 2 (12.94–10.85) points in MAE and RMSE, respectively, in bootstrap validation. Similar reductions in error were reproduced in sensitivity analyses under 10-fold cross-validation using penalized regression (LASSO and ridge) (LASSO λ1SE: MAE 12.43–10.47; RMSE 14.65–12.46). Because the FIM motor score is the sum of 13 items, even an overall reduction of approximately 2 points can appear as a 2-point difference in a single item or as an accumulation of small differences across multiple items. Consequently, this reduction in error may influence discharge support planning, including estimation of assistance needs, family education, and the selection of service intensity and welfare equipment; therefore, the error reduction observed in this study is considered to have clinical significance in supporting more reliable discharge-related decision-making.

These findings are consistent with the peak of spontaneous recovery and neuroplasticity during the early post-stroke phase (6–10 weeks after onset)^15,17)^ and support the usefulness of time-updated prediction incorporating longitudinal reassessment. Therefore, reassessment in this study may contribute to estimating assistance needs and planning discharge support, and the models may function as a decision-support tool based on dynamic prediction.

### Clinical interpretation of predictors in the admission model

In the Model 1, age, days since onset, BBS, the FIM motor score, and the FIM cognitive score were selected as predictors. All of these factors have been reported to be associated with ADL outcomes in previous studies^8–10,12,27–30)^, and the present findings support their validity.

First, age showed the largest standardized regression coefficient and was a major determinant of ADL outcomes in patients with stroke and low FIM at admission. With aging, muscle mass and strength decrease^31)^, neuroplasticity tends to decline^32,33)^, and the burden of comorbidities increases, potentially limiting improvements in motor learning capacity and activity levels^12)^. Kwakkel et al.^28)^ reported that the trajectory of recovery is largely determined during the first few weeks after stroke onset, and that inter-individual differences are strongly influenced by age, in addition to severity and lesion location. The present results also support this concept, positioning age as a physiological factor that limits recovery potential.

Next, days since onset reflects the timing of initiation of rehabilitation intervention, and a longer interval from onset to admission may delay ADL recovery because of the progression of disuse, muscle atrophy, and contracture. Early intervention after stroke is considered effective in promoting neuroplasticity and preventing learned nonuse^28)^, and in this study, patients with fewer days since onset at admission exhibited better ADL recovery. Thus, days since onset is an important temporal factor that influences recovery potential.

Additionally, the FIM cognitive score had the second largest standardized regression coefficient, and cognitive functions, including comprehension and memory, have been reported to contribute to motor learning effects^30)^. VanGilder et al.^30)^ stated that post-stroke cognitive impairment modifies responsiveness to rehabilitation, with reduced attention and executive function decreasing the efficiency of acquiring training effects. Therefore, cognitive function may act as a cognitive substrate supporting motor recovery through improved understanding of ADL task sequences and execution ability.

Although BBS showed the smallest standardized regression coefficient, it was the only physical function assessment index selected in the admission model. This suggests that even in patients with low FIM at admission (total FIM score at admission ≤55), quantitative assessment of balance function has some predictive value for ADL outcomes at discharge. The BBS reflects basic motor control, including sitting balance and standing stability, and can assess prerequisites for key ADL tasks such as walking and transfers. Mao et al.^27)^ have reported that the BBS has high reliability and validity in patients with stroke and shows a strong correlation with ADL measures. Louie and Eng^34)^ have also demonstrated that the BBS quantitatively reflects balance ability at admission and predicts independent walking after inpatient rehabilitation and the ability to walk in the community with high accuracy. Furthermore, it has been reported that the ability to maintain sitting posture predicts ADL improvement in patients with stroke and substantial ADL disability at admission^29)^, supporting the present findings. Therefore, the BBS, although simple, can be considered a clinically useful supplementary assessment for quantifying balance function at admission and understanding the level of physical function. However, in this sample, BBS scores were concentrated at low values (median [IQR], 3 [0–7]; 68.3% scored 0–5; 32.7% had BBS = 0), and interpretation of the BBS coefficient requires caution because of floor effects and restricted variability. In fact, although BBS was selected in penalized regression (LASSO λ1SE), the coefficient was small (standardized coefficient, 0.240). Therefore, in a low-FIM-at-admission population, it is reasonable to interpret BBS not as a strong discriminator but as a supplementary indicator that provides additional contribution beyond major predictors, including FIM and age.

Overall, in the Model 1, factors that constrain recovery potential, such as age and days since onset, and factors reflecting motor and cognitive functional levels, including BBS and FIM scores, appear to contribute in an integrated manner to the multidimensional prediction of ADL at discharge.

### Clinical implications of the 3-week model and the value of reassessment

In the Model 2, in addition to age, days since onset, and the FIM cognitive score at admission, the FIM motor score at 3 weeks after admission was selected as a key explanatory variable. Given that it had the largest standardized regression coefficient, these results indicate that the trajectory of early recovery in the initial post-stroke phase is a major determinant of subsequent ADL independence.

This finding is consistent with that of Veerbeek et al.^12)^, which suggested that early changes in motor function determine the slope of the ADL recovery curve, and supports the concept described by van der Groen et al.^15)^ that a single time-point assessment cannot fully capture inter-individual differences and that time-updated prediction using multiple time points improves predictive accuracy. Furthermore, the FIM motor score at 3 weeks after admission may represent a comprehensive recovery index reflecting not only motor ability but also responsiveness to rehabilitation interventions, activity tolerance, and adaptation to the living environment. However, the FIM motor score at 3 weeks is temporally close to the discharge outcome (i.e., a near-outcome predictor), and part of the improved predictive accuracy of Model 2 may be explained by this temporal proximity. Conversely, the mean length of stay in this study was 78.9 days, and reassessment at 3 weeks after admission occurred with an average of approximately 58 days (approximately 8 weeks) remaining until discharge; thus, the model did not simply incorporate an assessment conducted immediately before discharge. Because length of stay varies across individuals, reassessment may be closer to discharge in some short-stay cases, which should be considered when interpreting the results. Therefore, the central message of this study is not that future outcomes can be fully predicted at admission, but rather that updating predictions by incorporating the recovery course after admission—dynamic, time-updated prediction—has clinical value.

In fact, in penalized regression (LASSO, λ1SE), the FIM motor score at 3 weeks after admission showed the largest contribution in Model 2 (LASSO coefficient for standardized predictors: 0.860). In contrast, predictors, including the FIM motor score at admission, BBS, and FMA lower extremity, which had non-zero coefficients and were selected in Model 1 (LASSO), shrank to zero and were not selected in Model 2 at λ1SE. This suggests that the 3-week assessment captures and summarizes the results of early recovery and explains ADL at discharge with fewer predictors.

In this sample, the mean days since onset were 21.3 days at admission and approximately 42 days at the 3-week assessment, corresponding to the peak period of spontaneous recovery (6–10 weeks after stroke onset) reported by Kwakkel et al.^17)^ This period is considered a stage where neuroplasticity is most active and represents a critical window for recovery, during which spontaneous recovery and the effects of early intervention overlap. Therefore, reassessment at this timing is not merely follow-up observation but may be a clinically useful process that dynamically reflects recovery status and helps to concretize discharge support and goal resetting.

Based on these findings, a stepwise implementation may be reasonable in clinical practice: providing an initial, coarse estimate using Model 1 at admission and subsequently updating to Model 2 at 3 weeks to refine discharge support planning.

### Study limitations and future directions

This study had some limitations.

First, this was a single-center retrospective observational study, and the target population was limited to patients with low FIM at admission (total FIM score ≤55). Because our hospital has an acute-care unit, patients tend to transition to convalescent rehabilitation relatively early after stroke onset (mean, 21.3 days). The timing of transfer in this study was comparable to reports from other countries describing admission to a rehabilitation hospital at approximately 2–3 weeks after stroke onset (mean, 17.9 days)^35)^ and a representative report indicating transfer at approximately 20 days after onset^36)^. In contrast, cohort studies have also reported that the initiation of inpatient rehabilitation may be delayed to a mean of approximately 9.8 ± 6.7 weeks^37)^. Therefore, although the applicability of our models may be higher in patients who transition relatively early after onset, caution is required when generalizing the findings to settings where transfer occurs later. Future studies should evaluate external validity and generalizability using multicenter data with different transfer timings and healthcare systems. Additionally, to address potential overfitting, we performed not only internal validation using bootstrap resampling but also sensitivity analyses using penalized regression (LASSO and ridge) with 10-fold cross-validation, confirming that the superiority of Model 2 was reproduced after changing the analytical methods. However, the set of predictors and the estimated regression coefficients differed between the stepwise multiple linear regression and the penalized regression approaches (LASSO/ridge), suggesting that coefficient estimates may be sample-dependent and that model specification may not be stable across analytical methods. Accordingly, the generalizability of the present models is inherently limited. Moreover, these validations were conducted within the same dataset and do not guarantee performance in external populations; therefore, external validation using temporal split-sample validation and/or multicenter datasets is warranted. In addition, because floor effects (accumulation at 0) were observed for the BBS in this study, regression coefficients may become unstable for indicators with limited variance; thus, caution is needed when interpreting the contribution of the BBS and comparing models.

Second, although these multiple linear regression models predicted a continuous outcome (FIM motor score at discharge), binary decisions, including whether the patient will achieve a certain threshold at discharge, are frequently required in clinical practice. To enhance practicality, future research should examine the setting of cut-off values and develop prediction models for binary outcomes (e.g., logistic regression, decision tree analysis, or a scoring system).

Third, although the Model 2 showed high predictive accuracy, the assessment time point was 3 weeks after initiating rehabilitation, potentially limiting its use for decision-making immediately after admission. Therefore, it is necessary to examine the validity of a stepwise clinical application, where a simple prediction model is used at the initial stage, and a more accurate model is applied at reassessment.

Fourth, because the FIM motor score was included as an explanatory variable in model development, informational overlap with the outcome may occur, and careful interpretation is required from the perspective of model neutrality. However, this study did not aim to develop a strictly theoretical model but to create a prediction tool that can be used immediately in clinical settings, with practicality prioritized. Although the main conclusions remained consistent even when sensitivity analyses using penalized regression (LASSO/ridge) were conducted, future studies should compare alternative models excluding the FIM motor score, reassess structural validity using approaches including partial correlation analysis, and conduct external validation.

Fifth, the outcome in this study was limited to the FIM motor score at discharge and may not fully reflect the multidimensional aspects of overall ADL. Given that our models primarily focused on physical function, future research should consider using a comprehensive ADL outcome such as the total FIM score (all 18 items) and develop prediction models that reflect integrated recovery in both motor and cognitive domains.

## Conclusions

In this study, we developed and compared 2 prediction models for the FIM motor score at discharge in patients with stroke and low FIM at admission who were admitted to a convalescent rehabilitation ward: an admission-based model (Model 1) and a model incorporating information at 3 weeks after admission (Model 2). Model 2 showed better predictive performance and calibration than Model 1 in this single-center cohort, suggesting that incorporating reassessment information may improve time-updated prediction of discharge ADL outcomes. However, given the limited generalizability and the potential instability of model specification across analytical approaches, external validation using multicenter data (and, if necessary, model updating) is required before broader clinical application.

## Supplementary Material

Supplementary Table S1. Standardized coefficients selected by LASSO regression (λ1SE; non-zero coefficients).

## References

[1] Feigin VL, Stark BA, et al.: Global, regional, and national burden of stroke and its risk factors, 1990–2019: a systematic analysis for the Global Burden of Disease Study 2019. Lancet Neurol. 2021; 20: 795–820. 10.1016/S1474-4422(21)00252-0 PMC8443449 34487721

[2] Nakaizumi D, Uchiyama K, et al.: Combinations of independent motor Functional Independence Measure Items associated with home discharge in stroke patients in post-acute rehabilitation wards: an UpSet plot analysis. Cureus. 2024; 16: e73982. 10.7759/cureus.73982 PMC11658900 39703277

[3] Mitsuhashi T: History and present condition of kaifukuki rehabilitation services. J Clin Rehabil. 2022; 31: 300–308. (in Japanese)

[4] Aoki Y, Nakayama K, et al.: A randomized controlled trial on the effects of decision aids for choosing discharge destinations of older stroke patients. PLoS One. 2024; 19: e0272115. 10.1371/journal.pone.0272115 PMC10810461 38271437

[5] Ushiroyama Y, Matsufuji S, et al.: Association between providing guidance to patient’s family and return to home among patients in a severe condition at the time of admission to a convalescent rehabilitation unit. Jpn J Community-based Compr Phys Ther. 2025; 5: 43–49. (in Japanese with English abstract)

[6] Ministry of Health, Labour and Welfare (Japan): Nyūin iryō-tō no chōsa·hyōka bunkakai (FY2025 8th meeting); Shiryo-hen ② [Inpatient medical care survey and evaluation subcommittee: document compilation ②] 2025 Jul 31. https://www.mhlw.go.jp/content/10808000/001533223.pdf [in Japanese] (accessed December 19, 2025)

[7] Werner RA: Predicting outcome after acute stroke with the Functional Independence Measure. Top Stroke Rehabil. 1994; 1: 30–39. 10.1080/10749357.1994.11754032 27680953

[8] Jeong S, Inoue Y, et al.: Formula for predicting FIM for stroke patients at discharge from an acute ward or convalescent rehabilitation ward. Jpn J Compr Rehabil Sci. 2014; 5: 19–25.

[9] Tokunaga M, Taniguchi M, et al.: Assessment of the effects of factors in stroke rehabilitation using eight multiple regression analyses—An analysis of the Japan Rehabilitation Database—. Jpn J Compr Rehabil Sci. 2015; 6: 78–85.

[10] Tokunaga M, Sannomiya K: Explanatory variables to use in a multiple regression analysis to predict stroke patients’ motor FIM score at discharge from convalescent rehabilitation wards: an investigation of patients with a motor FIM score of less than 40 points at admission. Jpn J Compr Rehabil Sci. 2020; 11: 102–108.

[11] Koyama T, Matsumoto K, et al.: A new method for predicting functional recovery of stroke patients with hemiplegia: logarithmic modelling. Clin Rehabil. 2005; 19: 779–789. 10.1191/0269215505cr876oa 16250198

[12] Veerbeek JM, Kwakkel G, et al.: Early prediction of outcome of activities of daily living after stroke: a systematic review. Stroke. 2011; 42: 1482–1488. 10.1161/STROKEAHA.110.604090 21474812

[13] Lang CE, Bland MD, et al.: Assessment of upper extremity impairment, function, and activity after stroke: foundations for clinical decision making. J Hand Ther. 2013; 26: 104–114, quiz 115. 10.1016/j.jht.2012.06.005 PMC3524381 22975740

[14] Tokunaga M, Katsura K, et al.: Increasing the prediction accuracy of FIM gain by adding FIM improvement for one month from admission to the explanatory variables in multiple regression analyses. Jpn J Compr Rehabil Sci. 2017; 8: 16–20.

[15] van der Groen O, Ghosh M, et al.: Point of view on outcome prediction models in post-stroke motor recovery. Neurorehabil Neural Repair. 2024; 38: 386–398.

[16] Hori H, Sonoda S, et al.: Relationship between change in ADL during two weeks from admission and ADL at discharge in post-stroke patients admitted to Kaifukuki rehabilitation wards. Jpn J Compr Rehabil Sci. 2019; 10: 82–87.

[17] Kwakkel G, Kollen B, et al.: Impact of time on improvement of outcome after stroke. Stroke. 2006; 37: 2348–2353. 10.1161/01.STR.0000238594.91938.1e 16931787

[18] Cowley LE, Farewell DM, et al.: Methodological standards for the development and evaluation of clinical prediction rules: a review of the literature. Diagn Progn Res. 2019; 3: 16. 10.1186/s41512-019-0060-y PMC6704664 31463368

[19] Steyerberg EW, Harrell FE Jr.: Prediction models need appropriate internal, internal-external, and external validation. J Clin Epidemiol. 2016; 69: 245–247. 10.1016/j.jclinepi.2015.04.005 PMC5578404 25981519

[20] Collins GS, Dhiman P, et al.: Evaluation of clinical prediction models (part 1): from development to external validation. BMJ. 2024; 384: e074819. 10.1136/bmj-2023-074819 PMC10772854 38191193

[21] Ministry of Health, Labour and Welfare (Japan). Nichijyō Seikatsu Kinō Hyōka Hyōka no Tebiki [Guidelines for the Assessment of Activities of Daily Living]. https://www.mhlw.go.jp/file/06-Seisakujouhou-12400000-Hokenkyoku/0000038913.pdf [in Japanese] (accessed December 19, 2025).

[22] Harrell FE Jr.: Regression modeling strategies: with applications to linear models, logistic and ordinal regression, and survival analysis. 2nd ed, Springer, New York, 2015.

[23] Steyerberg EW, Vergouwe Y: Towards better clinical prediction models: seven steps for development and an ABCD for validation. Eur Heart J. 2014; 35: 1925–1931. 10.1093/eurheartj/ehu207 PMC4155437 24898551

[24] Draper NR, Smith H: Applied regression analysis. 3rd ed. Wiley, New York, 1998.

[25] Tibshirani R: Regression shrinkage and selection via the lasso. J R Stat Soc Series B Stat Methodol. 1996; 58: 267–288.

[26] Hoerl AE, Kennard RW: Ridge regression: biased estimation for nonorthogonal problems. Technometrics. 1970; 12: 55–67.

[27] Mao HF, Hsueh IP, et al.: Analysis and comparison of the psychometric properties of three balance measures for stroke patients. Stroke. 2002; 33: 1022–1027. 10.1161/01.str.0000012516.63191.c5 11935055

[28] Kwakkel G, Kollen B, et al.: Understanding the pattern of functional recovery after stroke: facts and theories. Restor Neurol Neurosci. 2004; 22: 281–299. 15502272

[29] Okamoto S, Sonoda S, et al.: Relationship between Functional Independence Measure (FIM) score on admission and influence of inhibitive factors in a comprehensive inpatient stroke rehabilitation ward. Jpn J Compr Rehabil Sci. 2018; 9: 59–65.

[30] VanGilder JL, Hooyman A, et al.: Post-stroke cognitive impairments and responsiveness to motor rehabilitation: a review. Curr Phys Med Rehabil Rep. 2020; 8: 461–468. 10.1007/s40141-020-00283-3 PMC7987128 33767922

[31] Grimmer M, Riener R, et al.: Mobility related physical and functional losses due to aging and disease - a motivation for lower limb exoskeletons. J Neuroeng Rehabil. 2019; 16: 2. 10.1186/s12984-018-0458-8 PMC6318939 30606194

[32] Popa-Wagner A, Hermann DM, et al.: The age-associated decline in neuroplasticity and its implications for post-stroke recovery in animal models of cerebral ischemia: the therapeutic role of extracellular vesicles. J Cereb Blood Flow Metab. 2026; 46: 222–235. 10.1177/0271678X251365020 PMC12331653 40770923

[33] Burke SN, Barnes CA: Neural plasticity in the ageing brain. Nat Rev Neurosci. 2006; 7: 30–40. 10.1038/nrn1809 16371948

[34] Louie DR, Eng JJ: Berg Balance Scale score at admission can predict walking suitable for community ambulation at discharge from inpatient stroke rehabilitation. J Rehabil Med. 2018; 50: 37–44. 10.2340/16501977-2280 29068037

[35] Shah S, Vanclay F, et al.: Efficiency, effectiveness, and duration of stroke rehabilitation. Stroke. 1990; 21: 241–246. 10.1161/01.str.21.2.241 2305399

[36] van Straten A, van der Meulen JH, et al.: Length of hospital stay and discharge delays in stroke patients. Stroke. 1997; 28: 137–140. 10.1161/01.str.28.1.137 8996502

[37] Paolucci S, Antonucci G, et al.: Functional outcome of ischemic and hemorrhagic stroke patients after inpatient rehabilitation: a matched comparison. Stroke. 2003; 34: 2861–2865. 10.1161/01.STR.0000102902.39759.D3 14615613

