# Supplementary material for: Time-updated Prediction of Discharge Functional Independence in Patients with Stroke and Low Functional Independence Measure in a Convalescent Rehabilitation Ward: Comparison between Admission and 3-week Models
Source: Phys Ther Res. 2026 Jun 4;29(2):133–42. doi: 10.1298/ptr.25-E10391 (PMC13624557; doi:10.1298/ptr.25-E10391)
Supplement: Supplementary file 1 [file ptr-29-133-s001.pdf]

Supplement Table S1. Standardized coefficients selected by LASSO regression (λ1SE; non-zero coefficients)

Model 1 (Admission model)

Predictor	Standardized coefficient (LASSO, λ1SE)
Pre-admission mRS	-1.510
FIM cognitive score at admission	0.786
FIM motor score at admission	0.615
Age	-0.452
BBS	0.240
Days from onset to admission	-0.159
FMA lower extremity	0.103

Model 2 (3-week model)

Predictor	Standardized coefficient (LASSO, λ1SE)
FIM motor score at 3 weeks	0.860
Pre-admission mRS	-0.528
Age	-0.296
FIM cognitive score at admission	0.207
Days from onset to admission	-0.060

Abbreviations: mRS, modified Rankin Scale; FIM, Functional Independence Measure; BBS, Berg Balance Scale; FMA, Fugl–Meyer Assessment.
